# Lexical and grammatical development in trilingual speakers of isiXhosa, English and Afrikaans

**DOI:** 10.4102/sajcd.v63i2.141

**Published:** 2016-05-20

**Authors:** Anneke P. Potgieter

**Affiliations:** 1 Department of General Linguistics, Stellenbosch University, South Africa

## Abstract

**Background:**

There is a dearth of normative data on linguistic development among child speakers of Southern African languages, especially in the case of the multilingual children who constitute the largest part of this population. This inevitably impacts on the accuracy of developmental assessments of such speakers. Already negative lay opinion on the effect of early multilingualism on language development rates could be exacerbated by the lack of developmental data, ultimately affecting choices regarding home and school language policies.

**Objectives:**

To establish whether trilinguals necessarily exhibit developmental delay when compared to monolinguals and, if so, whether this delay (1) occurs in terms of both lexical and grammatical development; and (2) in all three the trilinguals’ languages, regardless of input quantity.

**Method:**

Focusing on isiXhosa, South African English and Afrikaans, the study involved a comparison of 11 four-year-old developing trilinguals’ acquisition of vocabulary and passive constructions with that of 10 age-matched monolingual speakers of each language.

**Results:**

The trilinguals proved to be monolingual-like in their lexical development in the language to which, on average, they had been exposed most over time, that is, isiXhosa. No developmental delay was found in the trilinguals’ acquisition of passive constructions, regardless of the language of testing.

**Conclusion:**

As previously found for bilingual development, necessarily reduced quantity of exposure does not hinder lexical development in the trilinguals’ input dominant language. The overall lack of delay in their acquisition of the passive is interpreted as possible evidence of cross-linguistic bootstrapping and support for early multilingual exposure.

## Introduction

### Background

Currently, half of the world’s children are estimated to be growing up learning two or more languages due to the nature of the home and/or community contexts in which they are being reared (Grosjean, 2010). This phenomenon is especially common in Asia and Africa, including linguistically and culturally diverse South Africa, where high population density in the many low socio-economic status (SES) areas increases cross-linguistic contact. Despite the global pervasiveness of early multilingualism, there is a relative lack of studies on the effect of multilingual acquisition on linguistic development. This has led to divided opinions on the (dis)advantages of early multilingualism (Montanari, 2010, p. 103). Some argue that our ability for multilingual acquisition is part and parcel of our ‘human language making capacity’ (Meisel, as cited in Montanari, 2010, p. 103). Common lay opinion, however, largely holds that children growing up in multilingual contexts will necessarily suffer developmental language delay – this perhaps on grounds of anecdotal reports of children showing a (initial) delay in vocabulary development when their two or more languages are considered separately, and perhaps on grounds of many parents’ conviction that exposure to more than one language ‘confuses’ a young child.

### Objectives

On empirical grounds, the study aims to inform the above debate, the outcome of which has serious implications for child language professionals, child rearing practices and language-in-education policies. The second aim of the study is to contribute to the as yet extremely limited pool of information on developmental norms for speakers of Southern African languages, both monolingual and multilingual. Without such norms, speech-language pathologists cannot perform accurate developmental assessments of the linguistic abilities of the majority of South African children. In addition, where monolingual norms are available, they are often, for lack of another option, used for the assessment of bilingual children. As a result, an increasing number of bilinguals are being mistakenly diagnosed with specific language impairment (SLI) and word finding disorder, rather than credited as typically developing bilinguals who are likely to catch up to their monolingual peers, given sufficient time and exposure (cf. COST Action IS0804, n.d.; Paradis, 2010; Thordardottir, Rothenberg, Rivard & Naves, 2006). This misdiagnosis is largely driven by the fact that bilingual children typically have a smaller vocabulary size in each language than monolinguals do (Bialystok, Luk, Peets & Yang, 2010).

### Contribution to the field

Given the limitations of previous multilingualism research in that it focused mostly on bilinguals (cf., for example, Blom, 2010; Cornips & Hulk, 2008; Hoff *et al*., 2012; Hulk & Cornips, 2006; Unsworth, 2007, 2008), the study reported on here investigates firstly the added effect of a third language in the simultaneous acquisition process. In contrast to the purely observational methodology of many previous studies on trilingualism, the present study employs experimental tasks for data collection and involves a detailed analysis of linguistic data. Secondly, in investigating the simultaneous acquisition of isiXhosa, South African English (hereafter ‘English’) and Afrikaans, the focus is on a Germanic-Bantu combination of language families that has, to my knowledge, never been investigated in the context of early bilingual or trilingual language acquisition. Finally, the present study allows for the testing of Blom’s (2010) claim that when their weaker language in terms of exposure is considered, multilinguals will exhibit developmental delay, contrary to what the majority of studies on multilingual first language acquisition report regarding grammatical development in the input dominant language (cf., for example, Genesee, 2001; Meisel, 2001; Nicoladis & Genesee, 1997; Paradis & Genesee, 1996).

## The passive

### The acquisition of the passive

According to Deen (2011, p. 155), the passive might very well be the most widely researched grammatical construction in the field of child language acquisition due to the apparent delay in its acquisition. In the case of monolingual English children, research suggests that these learners generally take 5 years or longer to fully acquire the rules relating to passive constructions (Baldie, 1976; Demuth, Moloi & Machobane, 2010, p. 238; de Villiers & de Villiers, 1973). In the case of Dutch (the language from which Afrikaans largely derives), ‘hardly any’ uses of the passive have been noted in the speech of monolingual children of preschool age, that is, of 4 years and younger (Gillis & De Houwer, 1998, pp. 28, 35). Surprising then is the spontaneous use of the passive in the speech of children as young as 3 years that has been reported in the case of Southern African Bantu languages, including isiZulu (Suzman, 1985, 1987, 1990) and Sesotho (Demuth, 1989, 1990; Demuth *et al*., 2010); the Eastern Bantu languages Kiswahili and Kigiriama (Alcock, Rimba & Newton, 2011); and North American Inuit and Mayan languages (Demuth *et al*., 2010, p. 238).

Studies have shown that in those languages in which passive constructions are produced relatively early, adult speech generally exhibits a high percentage of such constructions (cf. Deen, 2002, for Kiswahili; Alcock *et al*., 2011, for Kigiriama; Kline & Demuth, 2008, for Sesotho). IsiXhosa is similar to Sesotho in terms of both language typology and the manner in which logical subjects may be questioned (the latter possibly increasing the frequency of passives – cf. Demuth *et al*., 2010). As such, given the reported age of acquisition of the passive by Sesotho monolinguals, it is highly likely that isiXhosa passives too are acquired earlier than English and Afrikaans passives in the case of monolingual children.

By choosing to investigate trilingual participants who are acquiring a combination of languages in which the passive is acquired at different rates among monolinguals, an enquiry into the possibility of cross-linguistic grammatical bootstrapping is made possible. If at an age at which isiXhosa monolinguals, but not yet English or Afrikaans monolinguals, have typically acquired the passive, isiXhosa–English–Afrikaans-developing trilinguals perform on par with or better than English and Afrikaans monolinguals, this would indicate that they may be using their more advanced knowledge of isiXhosa passives to support the development of the passive in their other two languages.

### A brief cross-linguistic comparison

The following brief description of passive constructions in the three languages of interest to this study will focus first on the two morphologically reduced Germanic languages, English and Afrikaans, before moving on to the highly agglutinating Bantu language isiXhosa. (For an in-depth comparative grammatical description of passives in these three languages, cf. Potgieter, 2014).

At the very least, the English and Afrikaans passive verbal sequence contains a passive participle, that is, a non-finite verb which encodes passive voice, and a free morpheme in the form of a passive auxiliary. In the case of regular English verbs, the passive participle is derived by attaching a passive morpheme in the form of the -*ed* or *-(e)n* suffix to the verb stem (Ouhalla, 1999, p. 170) as in *The cat was stroked (by the boy)*. In the case of regular Afrikaans verbs, this participle is derived by attaching the prefix *ge-* to the verb stem, as in *Die kat is (deur die seun) gestreel* (the Afrikaans translation of the English example). In English, the passive auxiliary BE can take various forms (i.e. *be*, *been*, *is* and *was/were*) depending on the tense or aspect that is being expressed. In a similar fashion, the Afrikaans passive auxiliary is phonetically realised as some form of WEES (‘be’), that is, as *wees, word*, *is* or *was*.

As illustrated in the examples above, an English passive sentence may optionally contain a *by*-phrase and an Afrikaans passive sentence a *deur*-phrase, where the complement of the preposition *by* or *deur* thematically corresponds to the expression functioning as the subject in the active counterpart of the sentence. Passives containing this type of phrase are often referred to as ‘long’ or ‘agentive’ passives, as opposed to ‘short’ or ‘agentless’ passives where the AGENT is left unspecified through the omission of this phrase. One function of the optional *by*- or *deur*-phrase is to place emphasis on a ‘heavy/lengthy’ AGENT argument (Ponelis, 1989, pp. 324–326).

Turning to isiXhosa, a sentence is marked as expressing the passive voice through the use of a bound morpheme that is attached to the verb stem. This affix commonly takes one of two forms: -*iw*- or -*w-*, the addition of the latter morpheme resulting in various (morpho-) phonological changes (Louw & Jubase, 1963, p. 111). Contrary to English and Afrikaans, isiXhosa does not indicate tense by means of a free morpheme in the form of a passive auxiliary. The agglutinating nature of isiXhosa verbal morphology renders the main verb finite in that tense is indicated by means of a specific affix on the verb itself. The affixes that mark the tense of passive isiXhosa verbs are generally the same ones also found with active verbs, except in the case of the perfective, which uses two distinct markers. Example (b) in (1) below illustrates the passive counterpart of the active sentence in (a):

**Figure:**
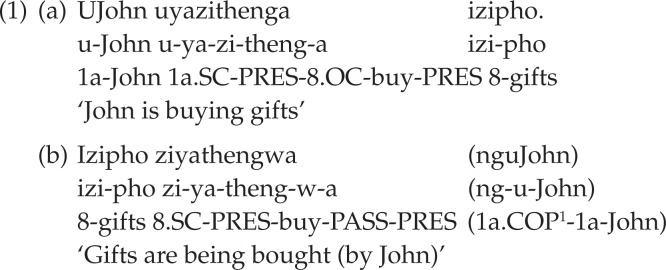


As shown in (1) above, isiXhosa allows for long passives through the use of a copular noun phrase (here, *nguJohn*) introduced by a copular prefix which serves the same semantic function as the English preposition *by* and the Afrikaans preposition *deur* in the context of passive sentences. The form of this prefix (here, *ng-*) is determined by the class of the noun to which it attaches (Louw & Jubase, 1963, p. 106). Although the isiXhosa copular noun phrase, like the English *by*-phrase, may occur only postverbally, its exact postverbal position may change in line with discourse factors (Du Plessis & Visser, 1992, p. 84).

Like English and Afrikaans, isiXhosa allows expletive passive constructions in which the structural subject position is thematically empty due to the object argument (in isiXhosa, regardless of it being definite or indefinite) remaining in its original position. In English and isiXhosa, the latter is a postverbal position, both languages being underlyingly SVO. In such constructions, the isiXhosa passive verb takes the expletive prefix *ku-* (Du Plessis & Visser, 1992, p. 70). Unlike in the other two languages, the structural subject position in isiXhosa expletive passives is not filled by a free morpheme (such as *there* or *daar*) but is left phonetically empty. Underlyingly, however, this position is filled by an existential pronominal element that is associated with *ku*- (Du Plessis & Visser, 1992, p. 72).^2^ An example of an isiXhosa expletive passive is given in (2) below, the definite object argument being italicised:

**Figure:**
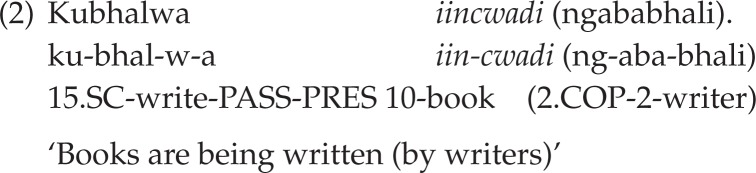


### Research questions

Given the objectives outlined in previous section, the primary research question that drove this study is: Does trilingual exhibit developmental delay when compared to monolingual? If so:

1. Does this delay occur both in terms of lexical and grammatical development (the latter being gauged on grounds of knowledge of passive constructions)?
2. Does this delay occur in the case of all three languages, or only in the language(s) that are weaker in terms of quantity of input?

## Research method and design

### Participants

A total of 41 four-year-old children were recruited for participation: 11 isiXhosa-English-Afrikaans developing trilinguals (mean age 54.1 months), 10 monolingual isiXhosa controls (mean age 55.2 months), 10 monolingual English controls (mean age 54.1 months) and 10 monolingual Afrikaans controls (mean age 51.2 months). Seven of the trilinguals were female and the remainder male, with each monolingual group being equally divided in terms of gender. All participants qualified as typically developing on grounds of parents’ answers to those questions in a language background questionnaire (LBQ) that enquired as to developmental milestones, a possible history of (periods of) hearing impairment and general concerns about the child’s linguistic development. All participants were from homes with low SES. (Cf. Potgieter & Southwood, 2016, for more information on how SES was calculated and for an assessment of the suitability of the lexical measure used in the present study for use with low SES individuals).

Among prospective trilingual participants, an age of first exposure before 4 years in the case of each of the child’s three languages (cf. Potgieter, 2014, for justification), as well as a reported ability to communicate meaningfully in all and only the three languages of interest to this study, were considered as selection criteria. As for the monolingual groups, the typical high level of sociolinguistic diversity of most low SES areas in the Western Cape made finding prospective participants with exclusive exposure to only one language nearly impossible. As such, participants were considered suitably monolingual if their parents reported that they are unable to speak and/or understand any language other than the one of interest to the extent that they can coherently converse in that language. Also, if an additional language was spoken in the home, exposure had to be limited enough to prevent the child from being able to regularly and spontaneously produce words in that language.

### Setting

The trilingual participants were sourced from eight different crèches (i.e. day-care centres) situated in townships and low-income informal settlements. These crèches mostly serve children from a range of different racial, cultural and linguistic backgrounds. As such, their child populations consist predominantly of English- and Afrikaans-speaking so-called coloured^3^ children; followed in number by isiXhosa-speaking black children; and then black children of immigrant descent, speaking other indigenous African languages as first language (L1), with mostly English as second language (L2). The levels of multilingualism in these groups vary, but the majority of the children are at least functionally bilingual, knowing either English or Afrikaans (to varying extents) as one of their languages. At the time of the study, the trilingual participants from these crèches were receiving exposure to at least two languages in the home environment and at least three in the community and/or crèche context (Afrikaans and/or English being the primary media of instruction at the relevant crèches).

The isiXhosa monolinguals were recruited from three crèches in a particular township and informally from a number of homes in a low-income suburban area; the English monolinguals from three crèches in a low-income suburban area; and all the Afrikaans monolinguals from the same crèche in a farmworker community.

#### Ethical considerations

Ethical clearance for the study was obtained from the Research Ethics Committee: Humanities (NHREC nr REC-050411-032) of Stellenbosch University. The objectives and procedures to be followed in the study were discussed verbally and presented in writing (as an informed consent form) to the parents of the child participants. The lack of financial compensation for participation and any hazard or direct benefit to participants was clearly communicated. Participants were assured that participation would be anonymous, voluntary and could be discontinued at any point. Signed informed consent from the parents and assent from the children were obtained (children indicating such assent with an ‘X’). All data have been protected either in a locked cabinet or, in the case of electronic data, on a password-protected laptop.

## Instruments, administration and scoring

### Language input

Information regarding the trilingual participants’ language exposure was gathered by means of a specially designed parental LBQ in conjunction with a teacher report (cf. Potgieter, 2014, for copies of these instruments) and analysed using the *Utrecht Bilingual Language Exposure Calculator* (UBiLEC; Unsworth, 2011a, 2011b, 2013). The LBQ mainly enquires as to the child’s language exposure over time and at the time of testing, within various contexts, and includes a section requiring an hour-by-hour description of a typical day in the child’s life (both during the week and over weekends), with specific information on the languages used in each context and their relative distribution. To control for varying literacy levels among the low SES respondents, the LBQ was administered as an oral interview conducted, in the case of the trilinguals, in the respondents’ homes and in the case of the monolinguals, mostly telephonically. The respondents’ L1 was employed in all cases, using a cultural and linguistic broker where necessary. All interviews were audio recorded, transcribed and (where necessary) translated. The results of teachers’ reports on the purpose and distribution of various languages in the crèche context were used to verify or correct parents’ reports on this matter.

The UBiLEC (Unsworth, 2011a, 2011b, 2013) consists of an LBQ and an accompanying Microsoft Excel spreadsheet that quantifies the collected exposure data, calculating values for the following four variables in the case of each language: (1) current amount of exposure (CAoE) as percentage of the child’s waking hours in a typical week; (2) cumulative length of exposure (CLoE) in years, accounting for varying amounts of exposure over time; (3) traditional length of exposure in years (TLoE), that is, age at testing minus age at onset of acquisition; and (4) quality of current input (in terms of ‘nativeness’, measured on a scale of zero to five). Although the regular UBiLEC LBQ was not employed in the present study, the specially designed LBQ elicited the necessary degree of detail concerning language exposure to render its results suitable for quantification via the UBiLEC spreadsheet.

### Lexical measure

The vocabulary tests employed in this study are the *Language Impairment in a Multilingual Society: Cross-linguistic Lexical Tasks-isiXhosa* (LITMUS-CLT-XHO; Southwood & Potgieter, 2013), *Cross-South African English* (LITMUS-CLT-SAE; Southwood, 2012b) and *Cross-Afrikaans* (LITMUS-CLT-AF; Southwood, 2012a). The aim of the research action behind the design of these instruments, and the 31 other language versions, was to disentangle bilingualism and SLI and to profile bilingual SLI in children from bilingual migrant communities in Europe (COST Action IS0804, n.d.). As such, the design of these instruments enables the fully comparable assessment of lexical ability across a multilingual child’s different languages (cf. Haman, Łuniewska & Pomiechowska, 2015).

The LITMUS-CLTs consist of four sections: noun production, verb production, noun comprehension and verb comprehension. The comprehension sections constitute a picture-selection task and the production sections a picture-naming task.

Testing was conducted in as quiet a room as possible on the crèche premises, except in the case of the few isiXhosa monolingual participants who did not attend the crèche and had to be tested at home. The trilingual participants were tested three times (once in each of their languages), with a week in between each testing. This period was deemed sufficient to minimise the chance of practice/priming effects. In addition, every effort was made to counterbalance the order of the language tests between children, taking into account the availability of assistants and participants, as well as crèche schedules. Practice/priming effects are, however, unlikely, given that the different language versions of the LITMUS-CLTs differ in their target word selection, the difficulty rating of words being the variable that was controlled for in their design. In the case of the monolingual participants, a single testing session for each child was necessary.

The order in which the four sections of the LITMUS-CLTs were administered was counterbalanced within each language group in the case of both the trilinguals and monolinguals. In the scoring of the production items, the soft score option (crediting regional variants and synonyms) was employed. See Potgieter and Southwood (2016) for more information on this decision, as well as some of the difficulties surrounding the scoring of responses to the isiXhosa version of this instrument.

### Grammatical measure

The instrument employed to test the acquisition of passive constructions is a subsection of Southwood and Van Dulm’s (2012a, 2012b, 2013) language therapy instrument known as *Receptive and Expressive Activities for Language Therapy* (REALt). This instrument was designed to enhance the language intervention process in the case of L1 and L2 English- and Afrikaans-speaking children with SLI or a language delay/disorder stemming from some other condition (Southwood & Van Dulm, 2012b, p. 1) and has since also been translated into isiXhosa. Included in the target population of this instrument are children from low SES communities whose general and classroom-relevant linguistic skills may be developed through the type of language stimulation that the use of this instrument can offer (Southwood & Van Dulm, 2012b, p. 1).

The passives subset of the REALt tests both the comprehension (by means of a picture-selection task) and production (by means of a sentence-completion task) of a number of different types of passive constructions, that is, short passives, long passives, actional passives, expletive passives and reversible long passives (i.e. long passives in which the expression denoting the animate AGENT argument and that denoting the animate THEME argument are interchangeable, even if such an alteration renders the interpretation somewhat improbable, for example, *The cat was chased by the dog* versus *The dog was chased by the cat*). In each testing session, the comprehension and production halves of the passive test were interchanged with the two halves of the vocabulary test to prevent the child from becoming bored with either test.

As the REALt was not designed to serve as a formal test instrument but as language therapy material, the researcher developed her own scoring system: a mark of one was awarded for every answer in the comprehension section that corresponded with the number of the target item, and a mark of zero for every incorrect answer. As for the production items, the child’s verbatim response was entered in a spreadsheet and a score of zero, one or two awarded, depending on the extent to which the response approached or deviated from the target answer. In short, an exactly on-target passive sentence or accurate passive sentence using a different yet suited passive verb was awarded two marks; a passive sentence with a morphological error on the verb or a long passive sentence that is on target except for an error in the agentive phrase was awarded a mark of one, and any answer that does not constitute a passive sentence was awarded a mark of zero. See Potgieter (2014) for a more complete description of the scoring process and the justification behind it.

## Results

### Results of the trilingual group’s language exposure measures

Table 1 presents, in the form of descriptive statistics, the results of the quantification of the trilinguals’ exposure data through means of the UBiLEC Excel spreadsheet. For the purposes of the present study, only the variables of CAoE and CLoE are reported on. To enable a comparison of CLoE across children of slightly varying ages (i.e. between 4.00 and 4.99 years), CLoE in years was recalculated to present a percentage portion of the child’s age in years. Results revealed that, at the time of testing, the majority of the trilinguals’ exposure was, on average, in the medium of English at 49.1%, followed by isiXhosa at 34% and then significantly less Afrikaans at 16.6%. However, by far the majority of the cumulative exposure that these children were exposed to over time took the form of isiXhosa at an average of 58.2%, with English and Afrikaans trailing behind at around 19% each.

**TABLE 1:** Trilingual group’s (*n* = 11) language exposure data, as percentage.

Variable	Language	M	SD
CAoE (as % of total exposure p/w)	isiXhosa	34	14.1
	English	49.1	14.3
	Afrikaans	16.6	8.6
CLoE (as % of age in years)			
	isiXhosa	58.2	22.2
	English	19.4	15.7
	Afrikaans	18.4	19.7

CAoE, current amount of exposure; CLoE, cumulative length of exposure; M, mean; SD, standard deviation.

### Results of the lexical measure

#### Descriptive statistics

The trilingual and different monolingual groups’ scores on the three different language versions of the LITMUS-CLT were not normally distributed; hence, median and IQR (rather than mean and range) have been used in the reporting of these data in Table 2.

**TABLE 2:** Monolinguals’ (*n* = 10 per language group) versus trilinguals’ (*n* = 11) median scores on the LITMUS-CLTs, as percentages with IQR below it.

Variable	isiXhosa		English		Afrikaans	
		
	Mono	Tri	Mono	Tri	Mono	Tri
**Comprehension**						
Nouns	71.88	71.88	84.38	56.25	84.38	56.25
	12.5 (68.8–81.3)	21.9 (59.4–81.3)	15.6 (81.3–96.9)	15.6 (65.6–81.3)	28.1 (68.8–96.9)	12.5 (50–62.5)
Verbs	62.5	59.38	73.44	53.13	73.44	53.13
	18.8 (50–68.8)	34.4 (37.5–71.9)	25 (56.3–81.3)	18.8 (43.8–62.5)	31.3 (59.4–90.6)	21.9 (37.5–59.4)
Total	67.97	62.5	79.69	54.69	79.69	54.69
	15.6 (59.4–75)	15.6 (57.8–73.4)	23.4 (65.6–89.1)	12.5 (56.3–68.8)	26.6 (64.1–90.6)	12.5 (46.9–59.4)
**Production**						
Nouns	43.75	40.63	73.44	15.63	70.31	25
	9.4 (37.5–46.9)	25 (28.1–53.1)	25 (56.3–81.3)	40.6 (15.6–56.3)	25 (56.3–81.3)	21.9 (9.4–31.3)
Verbs	29.69	31.25	68.75	12.5	43.75	15.63
	9.4 (25–34.4)	34.4 (9.4–43.8)	28.1 (34.4–62.5)	15.6 (9.4–25)	40.6 (40.6–81.3)	28.1 (3.1–31.3)
Total	35.94	34.38	71.88	18.75	54.69	20.31
	9.4 (31.3–40.6)	18.8 (23.4–42.2)	23.4 (48.4–71.9)	28.1 (12.5–40.6)	32.8 (48.4–81.3)	25 (6.3–31.3)
**Noun total**	**59.38**	**56.25**	**81.25**	**35.94**	**77.34**	**48.44**
	**12.5 (51.6–64.1)**	**17.2 (45.3–62.5)**	**18.8 (70.3–89.1)**	**26.6 (39.1–65.6)**	**26.6 (59.4–85.9)**	**10.9 (29.7–40.6)**
**Verb total**	**48.44**	**43.75**	**69.53**	**34.38**	**58.59**	**37.5**
	**12.5 (37.5–50)**	**28.1 (23.4–51.6)**	**25 (46.9–71.9)**	**15.6 (29.7–45.3)**	**32.8 (53.1–85.9)**	**25 (20.3–45.3)**
**Overall score**	**53.13**	**50**	**75.39**	**31.25**	**67.58**	**43.75**
	**13.3 (45.3–58.6)**	**15.6 (40.6–56.3)**	**23.4 (58.6–82)**	**18 (37.5–55.5)**	**29.7 (56.3–85.9)**	**7 (25–32)**

Across all three language groups, in both the monolingual and trilingual data, the total scores for comprehension are higher than the total scores for production, for example, 67.97% vs 35.94% in the isiXhosa monolingual data and 62.5% vs 34.38% in the isiXhosa trilingual data. This lag between the development of comprehension and production skills has been widely reported in the literature on child language acquisition and has been found to exist across many languages among both monolinguals and bilinguals. See, for example, Benedict (1979); Harrisa, Yeelesa, Chasina and Oakley (1995); Windsor and Kohnert (2004); and for studies on the acquisition of grammatical agreement by monolingual isiXhosa speakers, Gxilishe, Smouse, Xhalisa and de Villiers (2009) and Smouse, Gxilishe, de Villiers and de Villiers (2012). Also note that the scores on the noun sections are consistently higher than the scores on the verb sections. Consider, for example, the isiXhosa monolinguals’ noun total of 59.38% versus their verb total of 48.44% and the isiXhosa trilinguals’ noun total of 56.5% versus their verb total of 43.75%. These data align with a large body of studies that has shown the acquisition of nouns to precede the acquisition of other lexical categories across many languages, with some studies using bilingual participants (cf. Chan & Nicoladis, 2010, for references to numerous relevant studies).

#### Comparison across monolingual groups

The Kruskal–Wallis (non-parametric) ANOVA test was used to test for significant differences between the three monolingual groups’ overall test scores, total comprehension scores and total production scores ( *p* < 0.05 qualifying as significant). Results revealed a significant difference between the three language groups in terms of their overall test scores (*H*(2) = 9.11, *p* = 0.01). Bonferroni-adjusted post hoc tests revealed this to be due to the overall scores of the isiXhosa group (mdn = 53.1) being significantly lower than the overall scores of the English group (mdn = 67.6), *p* = 0.049, and the Afrikaans group (mdn = 75.4), *p* = 0.02. A second significant difference between the three language groups is found in the case of the total production scores (*H*(2) = 15.09, *p* < 0.01). This is again due to the isiXhosa group’s total production scores (mdn = 35.9) being significantly lower than those of the English group (mdn = 54.7), *p* < 0.01, and those of the Afrikaans group (mdn = 71.9), *p* < 0.01. In terms of total comprehension scores, however, the English (mdn = 80.5), Afrikaans (mdn = 79.7) and isiXhosa (mdn = 68) monolingual groups do not differ from one another significantly (*H*(2) = 4.39, *p* = 0.11). This overall pattern of results is also reflected in participants’ scores when the respective sections of the LITMUS-CLTs that test knowledge of verbs and nouns are considered in their own right, rather than subsumed under the overall score or total comprehension/production scores (cf. the descriptive statistics in Table 2).

#### Comparison between monolinguals and trilinguals

The non-parametric Mann–Whitney U test was used to test for significant differences between, in the case of each of the three languages, the monolingual and trilingual participants’ overall test scores, total comprehension scores and total production scores. In the case of both English and Afrikaans, the trilingual group (mdns: English = 43.8, Afrikaans = 31.3) scored significantly lower than the monolingual groups (mdns: English = 67.6, Afrikaans = 75.4) on the test as a whole (English: *Z* = −3.38, *U* = 6.5, *p* < 0.01; Afrikaans: *Z* = −3.63, *U* = 3; *p* < 0.01). On the comprehension sections of the English and Afrikaans tests too, the trilingual group (mdns: English = 62.5, Afrikaans = 54.7) was significantly outperformed (English: *Z* = −2.36, *U* = 21, *p* = 0.02; Afrikaans: *Z* = −2.92, *U* = 13, *p* < 0.01) by the monolingual groups (mdns: English = 80.5, Afrikaans = 79.7). Finally, this same pattern is found in the case of the production sections (English: *Z* = −3.45, *U* = 5.5, *p* < 0.01; Afrikaans: *Z* = −3.27, *U* = 8, *p* < 0.01), with the trilinguals (mdns: English = 20.3, Afrikaans = 18.8) faring significantly worse than the monolingual groups (mdns: English = 54.7, Afrikaans = 71.9).

In the case of isiXhosa, however, there were no significant differences between the trilingual and monolingual groups’ performance on any of the three compared measures, that is, not in terms of their overall test scores (*Z* = −0.67, *U* = 45; *p* = 0.5), total comprehension scores (*Z* = −0.67, *U* = 45; *p* = 0.5) or total production scores (*Z* = −0.49, *U* = 47.5, *p* = 0.62). This overall pattern of results is again also reflected in participants’ scores on the verb and noun subsections.

### Results of the grammatical measure

#### Descriptive statistics

Medians and IQRs are again applicable to the non-normally distributed scores on the passives subset of the three relevant language versions of the REALt, reported in Table 3.

**TABLE 3:** Monolinguals’ (*n* = 10 per language group) versus trilinguals’ (*n* = 11) median scores on the REALt, as percentages with IQR below it.

Variable	isiXhosa		English		Afrikaans	
		
	Mono	Tri	Mono	Tri	Mono	Tri
**Comprehension**						
Long actional passives/10	55	60	55	50	35	50
	20 (50–70)	40 (40–80)	30 (40–70)	20 (40–60)	30 (20–50)	30 (30–60)
Short actional passives/10	60	70	60	50	55	70
	40 (40–80)	20 (50–70)	30 (40–70)	20 (40–60)	30 (40–70)	30 (50–80)
Reversible passives/15	40	46.7	46.7	46.7	50	40
	26.7 (33.3–60)	26.7 (33.3–60)	20 (26.7–46.7)	20 (26.7–46.7)	6.7 (46.7–53.3)	20 (33.3–53.3)
Total/35	52.9	54.3	52.9	45.7	47.1	51.4
	22.9 (42.9–65.7)	11.4 (48.6–60)	17.1 (42.9–60)	8.6 (42.9–51.4)	22.9 (34.3–57.1)	11.4 (45.7–57.1)
**Production**						
Actional passives/10	55	70	0	0	0	0
	30 (40–70)	40 (35–75)	55 (0–55)	0 (0)*	35 (0–35)	0 (0)
Reversible passives/15	43.3	26.7	0	0	0	0
	43.3 (16.7–60)	40 (13.3–53.3)	26.7 (0–26.7)	3.3 (0–3.3)	10 (0–10)	0 (0)
Expletive passives/5	20	0	N/A	N/A	0	0
	80 (0–80)	20 (0–20)			30 (0–30)	0 (0)
Total/25	51	48	2	0	0	0
	38 (26–64)	34 (24–58)	38 (0–38)	2 (0–2)	22 (0–22)	0 (0)
**Overall score/60**	**50.4**	**49.2**	**33.3**	**26.7**	**30**	**31.7**
	**14.2 (42.5–56.7)**	**22.5 (37.5–60)**	**29.2 (25–54.2)**	**5.8 (25–30.8)**	**19.2 (20–39.2)**	**6.7 (26.7–33.3)**

Note: A single number in brackets indicates that all middle scores were at this number (here, at ‘0’)

Note firstly that, as in the case of the lexical measure, all participant groups generally have lower scores for production items than comprehension items. Consider, for example, the English monolinguals’ total production score of 2% versus their total comprehension score of 52.9% and the English trilinguals’ total production score of 0% versus their total comprehension score of 45.7%. Paradis (2010, p. 675) points out that production tasks are even more demanding for bilingual children than for monolingual children and cautions that such tasks may therefore produce an inaccurately poorer picture of bilinguals’ knowledge of a specific structure than is actually the case. Secondly, note that in the case of the trilinguals’ performance in each language, the median score for the comprehension section as a whole is close to 50%, the IQRs also being limited to no lower than 7% and no higher than 10% above 50%. The comprehension sections of the REALt present the child with a choice between three pictures – one picture being the target, one the opposer and the other the distractor. As such, a score of 33% for comprehension may be said to represent chance level. In order to ensure that the trilinguals’ scores for comprehension in each language, despite being relatively low, are still significantly higher than the chance level, a sample *t*-test was run on these data. Results confirmed that this was indeed the case for the isiXhosa (*t*(10) = 8.39, *p* < 0.01), English (*t*(10) = 4.72, *p* < 0.01) and Afrikaans (*t*(10) = 7.02, *p* < 0.01) data.

#### Comparison across monolingual groups

Care was taken in the design of the REALt to ensure that each test item, across the different language versions, targets the same structure and is comparable in terms of its degree of difficulty (Southwood & Van Dulm, 2012b, p. 4). However, this instrument was not designed with a statistical across-language comparison of results in mind; the statistical analysis reported below should therefore be interpreted with caution.

The Kruskal–Wallis non-parametric ANOVA test was used to test for, in the case of overall test scores and comprehension scores, significant differences between the three monolingual groups. Because the English and Afrikaans production data evidently suffered from floor effects (the median scores for all but one of the production subsections being 0%), these data were not deemed fit for statistical analysis. As such, the total production scores were compared in a descriptive fashion only. The ANOVA tests revealed a significant difference between the three language groups in terms of their overall test scores (*H*(2) = 7.55, *p* = 0.02). Bonferroni-adjusted post hoc tests revealed this to be due to the total scores of the isiXhosa group (mdn = 50.4), being significantly higher than the total scores of the Afrikaans group (mdn = 30), *p* = 0.02.

This significant difference in terms of overall test scores must be a result of differences in terms of production as the isiXhosa, English and Afrikaans monolingual groups do not differ significantly from one another in terms of their total comprehension scores (*H*(2) = 0.86, *p* = 0.65), their scores for the comprehension of long actional passives (*H*(2) = 5.5, *p* = 0.06) or their scores for the comprehension of short actional passives (*H*(2) = 0.11, *p* = 0.95). Recall that in the case of the LITMUS-CLT data too, the three monolingual groups did not differ significantly in terms of comprehension skills. It is clear from the raw median scores on the REALt, however, that the English and Afrikaans monolinguals fared much worse than the isiXhosa monolinguals in terms of their total production scores and their scores on all the production subsections.

#### Comparison between monolinguals and trilinguals

The participants’ percentage scores on the following test variables were statistically compared, using the Mann–Whitney U test: overall scores, total comprehension scores and scores for the comprehension of long and short actional passives, respectively. No significant differences were found in the case of any of the three languages (all U-values < 55 and all *p*-values > 0.17). Total production scores were again compared in a descriptive fashion only, using raw median scores. These scores were highly similar across the trilingual and relevant monolingual groups, that is, 48% vs 51% in the case of isiXhosa, 0% vs 2% in the case of English and 0% across both groups in the case of Afrikaans.

## Discussion

The results of the study are discussed below in the same order as which they were presented in above. To conclude the discussion, the research question (and sub-questions) presented in previous section are answered.

### Results of the lexical measure

#### Comparison across monolingual groups

The 4-year-old English and Afrikaans monolinguals in this study are on par with one another in terms of lexical development, but their isiXhosa monolingual counterparts seem to have significantly lower (at least productive) vocabulary skills. Possible explanations include: (1) a perhaps inevitable slight imbalance in the degree of difficulty of the LITMUS-CLT-XHO and that of the other two language versions; (2) culture-related differences between child-rearing practices and child-socialisation styles in black African versus so-called cape-coloured groups (the English and Afrikaans monolinguals belonging to the latter cultural group and the isiXhosa monolinguals to the former); and (3) despite a shared SES level, possibly lower print exposure among the ‘deep’ township isiXhosa monolinguals than among the English monolinguals from more suburban areas and among the Afrikaans monolinguals who live on farms but attend a crèche that has a small library.

#### Comparison between monolinguals and trilinguals

In terms of overall test scores, comprehension scores and production scores, the trilingual group fared significantly worse than the monolinguals on the English and Afrikaans tests but performed on par with the isiXhosa monolinguals. These findings are not surprising when considering the differing amounts of exposure that the trilingual versus monolingual groups have had to the respective languages. On average, the trilinguals have a CLoE to English that equates to only 19% of their lifetimes and their CAoE per week to English amounts to an average of not more than 49%. In the case of Afrikaans, the trilingual group’s average CLoE equates to only 18.4% of their lifetimes, and their average CAoE is only 16.6%. It is therefore not surprising that the trilingual group was consistently outperformed by the English and Afrikaans monolingual groups, who have a CLoE and CAoE to the respective languages of close to 100%.

The trilingual group average for CLoE to isiXhosa equates to 58.2% of their lifetimes and their CAoE to isiXhosa to 34%. This means that, with a CLoE to isiXhosa that is roughly three times that of their CLoE to other languages, but still nearly 40% less than that of monolinguals, the trilingual group was able to keep pace with their monolingual isiXhosa counterparts. Thus, the trilinguals in this study do show lexical developmental delay when compared to age-matched monolinguals, but only in the two languages that are weakest in terms of input quantity (measured in terms of CLoE), despite these trilinguals’ exposure to their strongest language also being significantly less than what monolinguals are privy to.

### Results of the grammatical measure

#### Comparison across monolingual groups

Recall that there was no statistically significant difference between the three monolingual groups in terms of their comprehension of passives, but that (on grounds of descriptive analysis) the English and Afrikaans monolinguals clearly fared much worse than the isiXhosa monolinguals in terms of production. This constitutes the exact opposite pattern of that found in the monolingual LITMUS-CLT data, in which the isiXhosa group was consistently outperformed by the English and/or Afrikaans group.

Let us assume that the significantly lower LITMUS-CLT scores among isiXhosa monolinguals are not a result of an imbalance in difficulty rating between the different language versions of the test. The opposing production patterns in the three monolingual groups’ lexical and grammatical test data may then be argued to indicate that, even if cultural child-rearing practices perhaps lower the quantity of child-directed input that the isiXhosa monolinguals receive, the negative effect of lowered quantity of exposure on the acquisition of passive constructions is cancelled out by the positive effect of the (assumed) higher frequency of such constructions in the isiXhosa child-directed speech that these children do receive. Recall from a previous section that the ability to produce passive constructions seems to emerge in English and Dutch monolinguals only after the preschool years, whilst the context-appropriate spontaneous use of the passive voice in the speech of children as young as three years has been reported for a number of other Southern and also Eastern Bantu languages (cf. Alcock *et al*., 2011; Demuth, 1989, 1990; Demuth *et al*., 2010; Suzman, 1985, 1987, 1990). Hence, the fact that the monolingual isiXhosa 4-year-olds in the present study seem to be more capable of producing passives than English and Afrikaans monolinguals is not surprising.

#### Comparison between monolinguals and trilinguals

The low median scores for both the trilingual and monolingual groups across languages, especially in the case of production, indicate the high degree of difficulty that passive constructions pose to the 4-year-old participants in this study (cf. Table 2). Importantly, however, despite receiving less exposure to all three of their languages, the trilingual group is managing to keep pace with the monolingual groups. This was shown statistically for comprehension and seems similar for production when raw median scores are considered. Recall that, in the case of the Afrikaans and English versions of the LITMUS-CLT, the trilinguals scored significantly lower than the monolinguals. It thus seems plausible that, at least as far as comprehension is concerned, the trilingual participants are transferring grammatical knowledge of passive constructions in isiXhosa (obtained through greater exposure to this language over time, perhaps coupled with the higher frequency of passives in Southern Bantu languages) to their knowledge of English and Afrikaans. If so, this would constitute a case of cross-linguistic grammatical bootstrapping, confirming that the assumed relatively high frequency of passive constructions in the input that a developing trilingual receives in one of her three languages (here, isiXhosa) can enhance its acquisition in her other two languages (here, Afrikaans and English).^4^

Considering the low median scores on the English and Afrikaans production sections, both the trilinguals and monolinguals scoring 0% on almost all subsections, it is evident that in the case of these two languages, the average four year old from a low SES context, even if monolingual, cannot yet produce passives. The bootstrapping effect mentioned above thus seems to be limited (at least at this stage in the trilinguals’ language development) to the comprehension of passives.

## Returning to the research questions

In light of the statistical comparison of the trilingual and monolingual groups’ test scores in each of the three languages and assuming that a significant difference indicates developmental delay, the simple answer to the question ‘Do trilinguals exhibit developmental delay when compared to monolinguals?’ is ‘Yes, but only in certain respects’.

Recall that sub-question (a) asked whether the developmental delay occurs both in terms of lexical and grammatical development. Results showed it to occur only in in the case of lexical development (in as far as lexical proficiency can be assessed by a vocabulary test) and not in the case of grammatical development (in as far as knowledge of passives may be taken as an indication of grammatical proficiency). Importantly, this lexical delay among trilinguals is seen to occur in the case of both production and comprehension across both nouns and verbs. Given the floor effects in both the monolinguals’ and trilinguals’ English and Afrikaans REALt production data, the reported absence of a delay in the trilinguals’ grammatical development is based on a statistical comparison of comprehension data only, although the raw production data show a similar trend.

The fact that the trilinguals do not exhibit developmental delay with regard to passives in any one of their three languages is an important, unexpected finding in light of the literature on bilingualism showing grammatical developmental delay to commonly occur in the case of the language of less exposure (cf., for example, Blom, 2010; Paradis, Nicoladis, Crago & Genesee, 2010). For a discussion of the differential effect of input on lexical versus grammatical development found in this study, see Potgieter (under review).

Sub-question (b) enquired as to whether the developmental delay among trilinguals manifests itself in all three languages or only in the language(s) that is weaker in terms of quantity of input. The answer here is that the developmental delay is only found in the case of the two languages to which the trilingual group, on average, received the least exposure over time, that is, English and Afrikaans. When the average amount of input that the trilingual group was receiving at the time of testing (i.e. CAoE rather than CLoE) is considered, the reason why there are significant differences between the trilinguals’ and monolinguals’ scores in the case of the English test, but not in the case of the isiXhosa test, is less clear – the trilinguals had a higher average CAoE to English compared to isiXhosa (and Afrikaans). Afrikaans, however, provides a simpler picture of the relationship between input and lexical development in being the language to which the trilinguals received the least exposure both in terms of CLoE and CAoE, and also the one in which the trilinguals exhibit the largest developmental delay.

Overall, the results of the present study align with the findings of a multitude of bilingualism studies in which participants’ lexical development lagged behind that of monolinguals in the case of their language of less exposure (cf., for example, MacLeod, Fabiano-Smith, Boegner-Page & Fontolliet, 2012; Thordardottir, 2011; Thordardottir & Brandeker, 2010, in press). The results further support those of bilingualism research in proving again that necessarily reduced exposure does not hinder lexical development in the input dominant language (in terms of exposure over time). Significantly, the present study has shown this to be the case even when input in the dominant language constitutes as little as 58.2% of multilingual participants’ overall language exposure over time (as opposed to the approximately 100% exposure monolinguals receive).

## Conclusion

The study has shown that, among low SES developing trilingual learners of isiXhosa, English and Afrikaans, necessarily reduced input does not lead to a delay in their acquisition of the passive in any of their three languages, or to a delay in lexical development in their input dominant language, isiXhosa. The lack of any delay in their acquisition of the passive could be a result of cross-linguistic bootstrapping in the form of transfer of advanced knowledge of passives in one language to their knowledge of passives in the other two languages. This finding contributes theoretically to our understanding of the interaction between multilinguals’ different language systems. It is also of practical value in indicating that exposing a child to multiple languages from a young age may support the earlier development of certain features in the child’s languages. In addition, the study has shown that it is possible for young children exposed to as many as three languages to attain a monolingual-like vocabulary in one language *plus* a certain amount of vocabulary in an additional two languages, a benefit that monolinguals are not privy to. Finally, in its assessment of both monolingual and trilingual speakers of isiXhosa, South African English and Afrikaans, the study has contributed to the limited pool of normative data on lexical and grammatical development among speakers of Southern African languages.

Despite the use of a larger number of participants that is typical of trilingualism research, the generalisability of the results of the study is admittedly limited by this number. Hopefully, this study has served to set the scene for larger scale research into multilingual language acquisition within the South African (and more broadly, the African) context. Only on grounds of sufficient research we may increase the accuracy of developmental assessments of children speaking local languages and ultimately cultivate full public understanding (specifically among parents and teachers) that childhood multilingualism does not necessarily pose a developmental hindrance but definitely offers children a valuable sociolinguistic skill – a skill that increases a child’s communicative and cultural resources and, in doing so, breaks down barriers.

## Footnotes

2 For (minimalist) generative analyses of the expletive construction in Bantu languages, cf. Zeller (2008).

3 In South Africa, this term is used to refer to persons of mixed ethnic origin, this mixed ancestry having roots in two or more of the following areas/groups: Europe, Asia and various indigenous Khoisan and Bantu tribes. Although this term is viewed as derogatory in certain contexts, no suitable alternative has unfortunately yet been established – hence the cautious use of the term here. The term ‘Black children’ is used with similar caution to refer to children of Bantu descent when specific ethnic/tribal affiliation is either irrelevant, or when wanting to refer to this group as a whole.

4 This type of implicit learning effect caused by the naturally or experimentally increased frequency of passive constructions in the input has already been reported to occur within a single language (rather than cross-linguistically). Specifically, increasing the frequency of passives in the input that English monolingual participants received led to their earlier comprehension of passives (cf. Bencini & Valian, 2008; Brooks & Tomasello, 1999; Huttenlocher, Vasilyeva, Cymerman & Levine, 2002; Huttenlocher, Vasilyeva & Shimpi, 2004; Savage, Lieven, Theakston & Tomasello, 2003) and to their earlier production of passives (cf. De Villiers, 1984; Vasilyeva, Huttenlocher & Waterfall, 2006; Whitehurst, Ironsmith & Goldfein, 1974).
